# Mechanisms and Predisposing Conditions for Statin‐Induced New‐Onset Type 2 Diabetes Mellitus: A Paradox Relative to Their Pleiotropic Metabolic Effects

**DOI:** 10.1002/edm2.70305

**Published:** 2026-08-12

**Authors:** Ali Nosrati Andevari, Mohsen Koolivand

**Affiliations:** ^1^ Department of Clinical Biochemistry, Afzalipour Faculty of Medicine Kerman University of Medical Sciences Kerman Iran; ^2^ Royan Stem Cell Technology Company Tehran Iran

**Keywords:** insulin, isoprenoid, SNARE, statin, T2DM

## Abstract

**Introduction:**

Type 2 diabetes mellitus (T2DM) is one of the most common metabolic disorders arising from decreased insulin secretion and insulin sensitivity. Patients with T2DM take statins to prevent diabetic complications and mitigate mortality risk associated with this disease. Statins primarily function by blocking HMG‐CoA, resulting in reduced cholesterol levels. Statins have been suggested to possess certain antidiabetic properties. However, the overall effects of statins remain controversial. The aim of this study was to investigate the mechanisms and triggering conditions for the development of statin‐induced T2DM, despite their reported pleiotropic antidiabetic effects.

**Methods:**

This paper is a narrative review. A thorough literature search was carried out across PubMed, Scopus and Google Scholar to identify studies relevant to the evaluation.

**Results:**

In the cholesterol biosynthesis pathway (the mevalonate pathway), in addition to cholesterol, isoprenoids such as farnesyl pyrophosphate and geranylgeranyl pyrophosphate are also produced. Inhibition of these metabolites largely mediates the diabetogenic action of statins. The effects of statins on insulin secretion appear to be largely independent of cholesterol. However, the inhibition of cholesterol synthesis in pancreatic beta cells reduces insulin secretion through impaired function of SNARE proteins and calcium channels. The most prominent effects of statins on insulin secretion and sensitivity are attributed to the suppression of isoprenoids, particularly Cdc42, Rac1 and Rab. Furthermore, studies have shown that statins directly affect the levels of some insulin‐sensitivity‐related factors, independent of the identification of isoprenoid‐mediated pathways.

**Conclusions:**

Atorvastatin, simvastatin and rosuvastatin possess the most pronounced diabetogenic properties. In contrast, lovastatin, fluvastatin, pitavastatin and most notably pravastatin exert either neutral or advantageous effects with respect to glucose metabolism.

## Graphical abstract

The primary function of statins is to inhibit cholesterol synthesis, which contributes to their antidiabetic effects. However, the majority of the diabetic effects of statins are due to inhibition of isoprenoid synthesis. Atorvastatin, simvastatin and rosuvastatin possess the most pronounced diabetogenic properties. In contrast, lovastatin, fluvastatin, pitavastatin and most notably pravastatin exert either neutral or advantageous effects with respect to glucose metabolism.

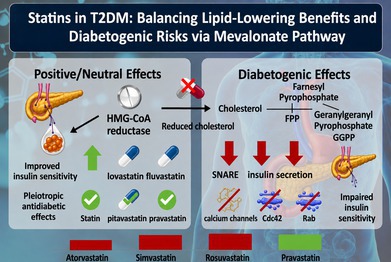

**Abbreviations**
ACC1: acetyl CoA carboxylase‐1
AKT: protein kinase B
ASK1: apoptosis signal‐regulating kinase 1
CAT‐1: carnitine acyltransferase‐1
CYP450: cytochrome P450
DAG: diacylglycerol
FDA: Food and Drug Administration
FPP: farnesyl pyrophosphate
G6Pase: glucose‐6‐phosphatase
GGPP: geranylgeranyl pyrophosphate
GLUT: glucose transporter
GSK‐3: glycogen synthase kinase 3
GSVs: GLUT4 storage vesicles
HFD: high‐fat diet
HMG‐CoA: 3‐hydroxy‐3‐methylglutaryl coenzyme A
HOMA‐β: homeostasis model assessment of β‐cell
HSkMCs: human skeletal muscle cells
IRS‐1: insulin receptor substrate‐1
mTORC1: mammalian target of rapamycin complex‐1
NLRP3: nod‐like receptor family pyrin domain containing 3
PAMPs: pathogen‐associated molecular patterns
PBMCs: human peripheral blood mononuclear cells
PCSK9: proprotein convertase subtilisin/kexin type 9
PEPCK1: phosphoenolpyruvate carboxykinase 1
PP2C: protein phosphatase 2C
PRRs: pattern recognition receptors
PTEN: phosphatase and tensin homologue
PXR: pregnane X receptor
SGK2: serum/glucocorticoid‐regulated kinase 2
SIRT‐1: sirtuin‐1
SNARE: soluble N‐ethylmaleimide‐sensitive factor attachment protein receptors
T2DM: type 2 diabetes mellitus
VAMP‐2: vesicle‐associated membrane protein 2

## 1 Introduction

Type 2 diabetes mellitus (T2DM) is a highly common metabolic disorder that affects how the body utilises glucose from the blood. T2DM arises from either reduced insulin sensitivity or insufficient insulin secretion. In recent years, the incidence of this disease has risen significantly as a result of lifestyle changes, increased obesity rates and sedentary behaviour [1, 2]. Type 2 diabetes mellitus (T2DM) is associated with both microvascular and macrovascular complications. The microvascular complications include nephropathy, retinopathy and neuropathy, whereas the macrovascular complications include cardiovascular diseases (CVDs) and cerebrovascular disease [3]. Statins are a class of medications that patients with T2DM take to prevent diabetic complications and mitigate T2DM‐attributable mortality risk [4]. Lowering cholesterol and low‐density lipoprotein cholesterol (LDL‐C) is a key function of statins. Elevated LDL‐C promotes the development of both microvascular and macrovascular complications. In this regard, studies have shown that reducing LDL‐C by about 39 mg/dL results in an approximately 25% reduction in CVDs. These incidents are progressively reduced with higher‐intensity statin therapy. Hence, physicians prescribe statins even for individuals with mild hyperlipidemia to prevent CVDs [5, 6, 7]. Nevertheless, recent studies have demonstrated that statin therapy may be associated with a higher risk of new‐onset T2DM [5, 8]. Statins indeed have antidiabetic effects as well. Based on available preclinical and clinical evidence, this study intends to examine the mechanisms and conditions that trigger statin‐induced new‐onset T2DM, despite these antidiabetic properties. The objective is to provide a foundation for physicians to manage statin therapy while balancing the risk of new‐onset T2DM.

## 2 Methods

The manuscript is a narrative review. We performed a comprehensive literature search across PubMed, Scopus and Google Scholar to find studies pertinent to the evaluation. Given the broad scope of this study and the aim to identify mechanisms linking statins to T2DM, articles published between 1999 and 2026 were included in this review. Studies conducted on cell lines, animal models and human subjects, including both original research and review articles, were incorporated into this manuscript.

## 3 Statins

The extensive efforts of Akira Endo led to the discovery of fungal inhibitors of cholesterol biosynthesis known as statins. Statins were first isolated as secondary metabolites of fungi such as *Penicillium citrinum*. Statins were first approved for clinical use by the United States Food and Drug Administration (FDA) in 1987. Lovastatin was the first marketed statin. Statins commonly used in clinical settings include lovastatin, atorvastatin, fluvastatin, simvastatin, pitavastatin, pravastatin and rosuvastatin. The primary action of statins is binding to the active site of 3‐hydroxy‐3‐methylglutaryl coenzyme A reductase (HMG‐CoA‐R), leading to its inhibition. HMG‐CoA‐R is a key enzyme in the mevalonate (cholesterol synthesis) pathway [9, 10, 11, 12]. Atorvastatin and rosuvastatin exhibit greater binding capacity for HMG‐CoA‐R, with rosuvastatin showing higher affinity than atorvastatin. Consequently, these two drugs are more potent inhibitors of the cholesterol synthesis pathway compared to other statins [13, 14]. Unlike other statins, pitavastatin and pravastatin are less potent at reducing cholesterol levels [15]. Notably, pitavastatin has a higher binding affinity for HMG‐CoA‐R than pravastatin and even simvastatin. Nevertheless, because simvastatin is used at higher doses than pitavastatin, it is more effective at lowering cholesterol levels. Overall, pitavastatin and rosuvastatin are newer than other statins. In addition, pitavastatin is available at lower doses on the market compared to other statins. This is due to the fact that pitavastatin has fewer drug interactions than other lipophilic statins (discussed below). Because it is minimally metabolised by cytochrome P450 (CYP450) isoenzymes, its potential for drug–drug interactions remains low. Consequently, pitavastatin has dosing restrictions in patients with mild to moderate liver impairment. However, it is not recommended for use in individuals with mild to moderate kidney impairment. Furthermore, pitavastatin is contraindicated in severe hepatic impairment, and the 4 mg dose should be avoided in patients with severe renal impairment [16, 17]. Hypercholesterolemia often occurs in T2DM patients, and atherosclerotic cardiovascular diseases (ASCVDs) represent the primary cause of death in these patients. As a result, statins attenuate the occurrence of CVDs and mortality related to them [10]. Statins are classified into lipophilic and hydrophilic types. The lipophilic statins (simvastatin, atorvastatin, lovastatin, fluvastatin, pitavastatin) can readily enter both hepatocytes and non‐hepatic cells. Hydrophilic statins (rosuvastatin, pravastatin) do not easily penetrate non‐hepatic cells. Pravastatin is more hydrophilic than rosuvastatin [18, 19]. With the exception of pravastatin and partially rosuvastatin, most statins undergo substantial first‐pass hepatic metabolism, resulting in a systemic bioavailability of only 5%–30% of the administered dose. Statins are predominantly metabolised by CYP450 enzymes. Pravastatin and rosuvastatin undergo the least metabolic processing [20]. Atorvastatin, simvastatin and lovastatin are predominantly metabolised by CYP3A4 (the most prevalent hepatic CYP450 isoenzyme), exhibiting a high potential for drug interactions. Conversely, fluvastatin, pitavastatin, pravastatin and rosuvastatin are minimally metabolised by CYP3A4. In general, due to the extensive hepatic metabolism of atorvastatin, lovastatin and simvastatin, their bioavailability is lower than that of other statins [21, 22]. Fluvastatin is extensively metabolised by CYP2C9, which has lower hepatic abundance than CYP3A4. Consequently, fluvastatin exhibits fewer drug interactions than atorvastatin, simvastatin and lovastatin. Although the difference may not be considerable, atorvastatin generally and at high doses confers a higher risk of hepatotoxicity relative to simvastatin [21, 23]. Thus, lipophilic statins are associated with more adverse effects than hydrophilic statins. A transient elevation of hepatic enzymes may occur in statin users, though it lacks clinical significance [22]. One of the factors influencing the efficacy, toxicity and side effects of statins is their half‐life. Atorvastatin and rosuvastatin have the longest half‐life (20 h), while simvastatin has about 12 h, pitavastatin 10 h, and the remaining statins have a half‐life of approximately 4 h. Statins are generally safe and well tolerated. Nevertheless, statin therapy, especially with long‐term exposure to these drugs, is sometimes associated with increased liver enzyme levels and the development of myopathy [20, 24]. Recently, much attention has been paid to other potential side effects of statins, especially the risk of developing T2DM [20]. In March 2012, the FDA, based on studies of patients using statins for the prevention of CVDs, warned that statin intake is associated with increased levels of fasting blood glucose (FBS) and glycosylated haemoglobin (HbA1c) [20, 25, 26]. Nevertheless, as previously noted, statins can activate mechanisms that contribute to a reduction in the incidence of T2DM and its associated complications. Overall, statins may influence the development of T2DM through their effects on insulin secretion and insulin sensitivity.

### 3.1 Statin‐Induced Reduction in Insulin Secretion via Inhibition of the Mevalonate Pathway

#### 3.1.1 The Connection Between Blocking Cholesterol Synthesis and Lowering Insulin Secretion

As previously noted, statins, particularly lipophilic statins, readily penetrate non‐hepatic cells. Higher doses of statins in these cells can greatly mitigate intracellular cholesterol. SNARE proteins (soluble N‐ethylmaleimide‐sensitive factor attachment protein receptors) play an important role in mediating fusion of insulin granules with the plasma membrane. Vesicle‐associated membrane protein 2 (VAMP‐2) is a SNARE protein located on vesicles; therefore, it is called a v‐SNARE. In contrast, syntaxin 1A and synaptosomal‐associated protein 25 (SNAP‐25) are SNARE proteins located on the plasma membranes of target cells and are thus referred to as t‐SNAREs. The binding of SNARE proteins on vesicles (v‐SNAREs) to SNARE proteins on the plasma membrane (t‐SNAREs) facilitates the process of exocytosis and the fusion of secretory granules with the plasma membrane [27]. Cholesterol is a major component of lipid rafts found in both the plasma membrane and secretory vesicles [28]. The proper localisation of SNARE proteins to the plasma membrane and secretory vesicles is dependent upon cholesterol. Consequently, decreased cholesterol levels in the plasma membrane and secretory vesicles impair the localisation and interaction of SNARE proteins, ultimately resulting in reduced insulin secretion. Furthermore, SNARE proteins participate in the transport of ion channels associated with insulin secretion, including voltage‐gated calcium channels, to the plasma membrane. Given that ion channels reside within the plasma membrane, an attenuation in cholesterol levels in membrane domains impairs the proper localisation of these channels. Consequently, based on these observations, inhibition of cholesterol synthesis in pancreatic beta cells mitigates insulin secretion through diminished function of both SNARE proteins and calcium channels [27, 29] (Table 1). Overall, elevated plasma LDL‐C levels in hypercholesterolemic patients with T2DM play a significant role in pancreatic beta‐cell function. To such an extent that increased cellular cholesterol disrupts beta‐cell function, proliferation and survival. As previously mentioned, statins lower plasma LDL‐C levels [30]. Of course, under hypercholesterolemic conditions, it is unlikely that intracellular cholesterol levels within beta cells would be mitigated to such an extent that it leads to a notable decrease in insulin secretion. Moreover, statins possess effects that increase insulin secretion through modulation of cholesterol metabolism. Cholesterol accumulation in beta cells impairs insulin secretion [31]. Statins also raise blood proprotein convertase subtilisin/kexin type 9 (PCSK9) levels. In fact, by decreasing intracellular cholesterol, they lead to increased expression of PCSK9. The primary function of PCSK9 is to promote the degradation of the low‐density lipoprotein receptor (LDLR), consequently decreasing the cellular uptake of LDL‐C [10, 32]. Thus, PCSK9 blocks cholesterol accumulation within beta cells [33]. These findings demonstrated that statins enhance insulin secretion through a reduction in plasma LDL‐C levels and an elevation in PCSK9 levels. Consequently, the cholesterol metabolism‐dependent effects of statins that attenuate insulin secretion are likely minimal.

**TABLE 1:** Mechanisms underlying statin‐induced impairment of insulin secretion.

Inhibition of cholesterol synthesis	Inhibition of isoprenoid synthesis	Other roles
Reduced function of SNARE proteins	Impaired dolichol biosynthesis	GLUT2 downregulation
Decreased calcium channel activity	Decreased activity of Cdc42, Rac‐1 and Rab GTPases	Voltage‐gated calcium channel mRNA and protein downregulation
	Impaired coenzyme Q10 biosynthesis	Decreased intracellular ATP levels
	Potassium and calcium channel dysfunction	Increased levels of long‐chain acyl‐CoA

Abbreviations: GLUT2, glucose; SNARE, soluble N‐ethylmaleimide‐sensitive factor attachment protein receptors.

#### 3.1.2 The Association Between Inhibition of Isoprenoid Synthesis and Decreased Insulin Secretion

The cholesterol biosynthesis pathway produces not only cholesterol but also isoprenoids like farnesyl pyrophosphate (FPP) and geranylgeranyl pyrophosphate (GGPP). Moreover, within this pathway, FPP also increases the production of dolichol. Consequently, beyond inhibiting cholesterol synthesis, statins suppress the synthesis of such isoprenoids and dolichol. FPP and GGPP are required for the post‐translational prenylation and subsequent activation of G‐proteins [20, 34]. It has been shown that G‐proteins play a role in insulin secretion. Specifically, Cdc42, Rac‐1 and Rab are essential for the formation of insulin secretory vesicles. These G‐proteins require GGPP for activation. Hence, isoprenylation of these G‐proteins is essential for the exocytosis of insulin‐containing granules [35, 36]. Ubiquinone, also called coenzyme Q10 (CoQ10), is another compound that plays a role in the insulin secretory pathway. The production of CoQ10 is dependent on GGPP. This compound is a constituent of the mitochondrial electron transport chain and plays a critical role in the generation of adenosine triphosphate (ATP). Given that ATP production is necessary for insulin secretion, an attenuation in CoQ10 levels may result in diminished insulin secretion [37]. Glucose is the most important signal for insulin secretion. Within the insulin secretory pathway, glucose is transported into pancreatic beta cells via the GLUT2 glucose transporter. Once inside these cells, glucose is phosphorylated to glucose‐6‐phosphate by the enzyme glucokinase. Subsequent metabolism of glucose results in ATP production. An elevation in the cytoplasmic ATP/ADP ratio induces closure of ATP‐sensitive potassium channels, resulting in membrane depolarisation (the membrane potential becomes more positive). This depolarisation subsequently opens voltage‐gated calcium channels, permitting calcium influx into the cell and consequently increasing intracellular calcium levels. The resulting increase in intracellular calcium concentration then stimulates exocytosis of insulin‐containing granules [38, 39]. Studies have shown that one of the effects of statins in reducing insulin secretion is their direct or indirect effect (through inhibition of the mevalonate pathway) on calcium channels [11]. Based on these findings, inhibition of isoprenoid synthesis can attenuate insulin secretion through impaired prenylation of the G‐proteins Cdc42, Rac1 and Rab, as well as through reduced CoQ10 production. Of course, statins may also exert direct effects on insulin secretion (Table 1).

#### 3.1.3 Studies Investigating the Impact of Statins on Insulin Secretion

Findings from multiple studies support the mechanisms described above. In a study by Zhao et al., both atorvastatin and pravastatin were shown to attenuate GLUT2 expression in human pancreatic islet beta cells. Glucose uptake levels in these cells after treatment with 100 nM statins were as follows: 58.76% (atorvastatin), 60.21% (pravastatin), 72.54% (rosuvastatin) and 89.96% (pitavastatin). Furthermore, these statins inhibited insulin secretion in a dose‐dependent fashion. Consequently, atorvastatin and pravastatin had a greater effect on reducing insulin secretion compared to the other statins [40]. In a study conducted by Zhou and colleagues on the mouse MIN6 cell line, simvastatin treatment inhibited insulin secretion by reducing cellular ATP levels, decreasing mRNA and protein expression of voltage‐gated calcium channels and GLUT2, and mitigating calcium influx [41]. Furthermore, Bellia et al. demonstrated that 1 year of treatment with simvastatin and rosuvastatin (20 mg/day) in patients with T2DM resulted in a significant decrease in the homeostasis model assessment of β‐cell function (HOMA‐β), consequently leading to impaired insulin secretion and a substantial elevation in HbA1c. However, insulin sensitivity remained unchanged [42]. Although simvastatin was given at a low dose and rosuvastatin at a moderate dose, the long duration of treatment had a negative effect on insulin secretion and HbA1c levels. However, insulin sensitivity was not altered. In another study, Yada and colleagues showed that simvastatin suppressed insulin secretion via inhibition of voltage‐gated calcium channels and subsequent attenuation of calcium influx into pancreatic beta cells. In contrast, pravastatin had no effect on calcium channels, calcium influx, or insulin secretion [43]. This result was consistent with the fact that pravastatin is more hydrophilic than simvastatin and that it has a weaker inhibitory effect on the mevalonate pathway relative to simvastatin. To complement the previous study, Curry et al. utilised mouse pancreatic beta cells and demonstrated that simvastatin exposure suppressed oxidative respiration as well as mitochondrial electron transport chain complexes in healthy cells. Moreover, this lipophilic agent inhibited the glucose‐induced activation of voltage‐gated calcium channels [38]. Several previous studies have examined the relationship between lovastatin and impaired insulin secretion. For instance, in a study by Tsuchiya and colleagues using MIN6 β‐cells and rat islets, lovastatin impaired the formation of insulin secretory granules and subsequently attenuated insulin secretion by inhibiting cholesterol and GGPP synthesis [44]. Consistent with earlier findings, Salunkhe et al. demonstrated that exposing INS‐1832/13 cells to rosuvastatin diminished glucose‐stimulated insulin secretion via inhibition of calcium channels, and these effects were reversed by the addition of mevalonate, indicating that isoprenoids play a critical role in calcium release and subsequent insulin secretion [45].

Inhibition of HMG‐CoA reductase results in elevated levels of acetyl CoA and acetoacetyl CoA. This elevation increases long‐chain Acyl‐CoA concentrations, which in turn activates ATP‐sensitive potassium channels and decreases insulin secretion [46]. Nevertheless, additional studies are required to substantiate the effects of statins through the aforementioned pathway.

We have outlined the mechanisms by which statins attenuate insulin secretion. However, we also discussed the positive relationship between statins and insulin secretion. One way statins beneficially affect beta‐cell function and insulin release is by reducing cellular stress. In a study by Chen et al., atorvastatin preserved pancreatic beta‐cell function in obese mice through enhanced beta‐cell proliferation and amelioration of endoplasmic reticulum stress [47].

### 3.2 The Association Between Statins and Insulin Resistance

#### 3.2.1 Inhibition of Cholesterol Synthesis

Lipophilic statins possess a high affinity for the plasma membrane. Consequently, they readily penetrate cells and may become involved in pathways associated with the development of insulin resistance [18, 48]. Statins induce insulin resistance and the development of T2DM in insulin‐sensitive cells, including adipocytes and skeletal muscle cells, via multiple pathways.

As previously noted, statins inhibit cholesterol synthesis [48]. Moreover, inhibition of cholesterol synthesis leads to a reduction in cholesterol levels within the plasma membrane [27]. Cholesterol is a major component of lipid domains in the plasma membrane and plays an important role in the signalling pathway initiated by membrane receptors [49]. Alterations in plasma membrane cholesterol levels impair the function and proper localisation of membrane receptors, including insulin receptors (IRs). Consequently, decreased membrane cholesterol levels may induce insulin resistance [48]. In a study by Sanvee et al., exposure of C2C12 myotubes to simvastatin resulted in dysfunction of IRs [50]. Dolichol may play a role in insulin sensitivity. As an important factor in N‐linked glycosylation, dolichol is essential for the proper processing of intracellular membrane‐bound receptors. Lovastatin had been demonstrated to significantly attenuate dolichol levels in mice [20].

#### 3.2.2 Downregulation of Isoprenoids and Key Factors Involved in Insulin Signalling

Rab‐4 is a protein associated with GLUT4 storage vesicles (GSVs) and is essential for the translocation of these vesicles to the plasma membrane [8, 51]. Rheb is another G protein that, like Rab‐4, requires isoprenoids for its intracellular activity. FPP is responsible for activating Rheb [52]. Rheb is a constituent of the mTORC1 (mammalian target of rapamycin complex‐1) complex within the insulin signalling cascade. Activation of this pathway results in the upregulation of GSV‐associated proteins, including GLUT4 and sortilin [53]. In a study by Li et al., exposure of C2C12 myotubes to simvastatin resulted in suppressed phosphorylation of the IR, IR substrate‐1 (IRS‐1) and protein kinase B (AKT); decreased expression of IR and IRS‐1; and impaired Rac1 activity. These changes led to reduced glucose uptake and diminished GLUT4 translocation to the plasma membrane. Notably, all of these effects were reversed by mevalonolactone, a mevalonate pathway intermediate [54]. In a study reported by Takaguri and colleagues, exposure of 3 T3‐L1 adipocytes to atorvastatin resulted in inhibition of the active form of Rab4 at the plasma membrane. Furthermore, atorvastatin diminished insulin‐stimulated tyrosine phosphorylation of IRS‐1 and serine/threonine phosphorylation of AKT. In contrast, pravastatin did not affect insulin signalling [55]. In another study, Ganesan et al. showed that treatment of 3 T3‐L1 cells with simvastatin led to decreased GLUT4 expression in these cells. However, treatment with pravastatin had no significant effect on GLUT4 expression [56]. Moreover, Yaluri et al. demonstrated that exposing L6 muscle cells to simvastatin attenuated the phosphorylation of IR, IRS‐1, AKT and glycogen synthase kinase 3β (GSK‐3β), along with GLUT4 expression, under both basal and insulin‐stimulated conditions. These effects were reversed by GGPP supplementation. In contrast, pravastatin treatment did not affect glucose uptake in these cells [57]. Furthermore, Jiang et al. reported that exposure of primary cultured rat cardiomyocytes to atorvastatin diminished mRNA and protein levels of GLUT4 and IRS‐1. In contrast, treatment with pravastatin and rosuvastatin did not significantly affect the expression of these factors [58]. Furthermore, Chamberlain and colleagues showed that treatment of 3 T3‐L1 adipocytes with lovastatin led to downregulation of the GLUT4 glucose transporter and induction of insulin resistance through inhibition of isoprenoid synthesis [59]. In another study, Zhao et al. reported that atorvastatin, pravastatin and rosuvastatin attenuated the levels of GLUT4, phosphorylated AKT (p‐AKT) and phosphorylated GSK‐3β (p‐GSK‐3β) in human skeletal muscle cells (HSkMCs) [40]. In addition, in a study conducted by Grunwald et al., eight patients without T2DM received various statins (simvastatin 20 mg/day, atorvastatin 10 and 40 mg/day, pravastatin 5 and 10 mg/day) for a period exceeding 6 months. Skeletal muscle biopsy specimens were collected from the patients. Additionally, the short‐term effects of rosuvastatin and simvastatin were investigated in vitro using glucose‐ and insulin‐stimulated human primary myotubes. 5′‐adenosine monophosphate–activated protein kinase (AMPK) and AKT play critical roles in cellular glucose uptake. AMPK activity was markedly increased in skeletal muscle cells following both long‐term and short‐term statin treatment. In contrast, AKT activity and protein levels were substantially attenuated. Furthermore, statins downregulated GSK3 and mTOR protein expression exclusively in vitro. Therefore, the sustained inhibition of AKT activation caused by statins may result in long‐term metabolic inflexibility and decreased insulin sensitivity [60]. In a study conducted by Sun and colleagues on high‐fat diet (HFD)‐fed mice and C2C12 cells, treatment with atorvastatin (6 and 12 mg/kg) for 22 days, in contrast to pravastatin (12 mg/kg), inhibited GLUT4 translocation to the plasma membrane in both C2C12 cells and HFD mice, leading to impaired glucose uptake. However, adding cholesterol and mevalonic acid reversed these results. Additionally, there was a significant positive correlation between free cholesterol levels in muscle tissue and GLUT4 protein expression on the muscle membrane. As previously noted, cholesterol plays a critical role in regulating metabolism within the plasma membrane and secretory vesicles, and isoprenoids also have important functions in increasing insulin sensitivity. Moreover, atorvastatin did not affect total GLUT4 protein expression in muscle. Due to the greater lipophilicity of atorvastatin compared to pravastatin, cellular uptake of atorvastatin by C2C12 cells was significantly higher than that of pravastatin. Furthermore, atorvastatin exhibited a longer half‐life and greater potency in inhibiting the mevalonate pathway relative to pravastatin [61]. In a study by Poletto et al., the experimental conditions differed from those of the earlier study on HFD‐fed mice. Obese mice were treated with atorvastatin (0.1% w/w) for 4 weeks, which resulted in increased GLUT4 expression and enhanced insulin sensitivity in adipose tissue. Notably, however, GLUT4 translocation to the cell surface was not assessed in this study [62]. In another study that examined the differences between two statins under preclinical conditions, Cho et al. reported that following 12 weeks of treatment, fasting blood glucose levels in HFD‐fed mice receiving pitavastatin were significantly lower compared to those receiving rosuvastatin [63]. Considering earlier studies, this result may be due to differences in the intensity of mevalonate pathway inhibition among statins, as well as differences in their half‐life [64]. As mentioned earlier, diminished cholesterol levels play an important role in insulin resistance‐related processes. Nevertheless, given the hydrophilic properties of rosuvastatin, it might be anticipated that it would not exert a significant negative effect on the insulin signalling pathway.

##### 3.2.2.1 Downregulation of Secondary Mediators Involved in Insulin Signalling

De las Heras and colleagues published a study showing that rosuvastatin (15 mg/kg/day) for 7 weeks in HFD‐fed mice mitigated leptin and increased sirtuin‐1 (SIRT‐1), PGC‐1α (peroxisome proliferator‐activated receptor gamma coactivator 1‐alpha), PPAR‐γ (peroxisome proliferator‐activated receptor gamma) and GLUT4 in adipose tissue. These rosuvastatin‐related findings improve insulin sensitivity in these mice [65]. Moreover, a study analogous to the previous one was performed by Valero‐Muñoz et al., demonstrating that rosuvastatin possesses the potential to enhance insulin sensitivity via its effects on leptin, SIRT‐1, PPAR‐γ and GLUT4 [66]. As previously mentioned, one of the factors involved in increasing insulin sensitivity is sortilin. In a study published by Andevari et al., treatment with atorvastatin (40 mg/day) for 3 months in patients with T2DM attenuated serum sortilin levels. Importantly, these patients had no prior history of statin use, and by the end of the study, their glycemic profile had improved significantly relative to baseline [48].

**FIGURE 1:**
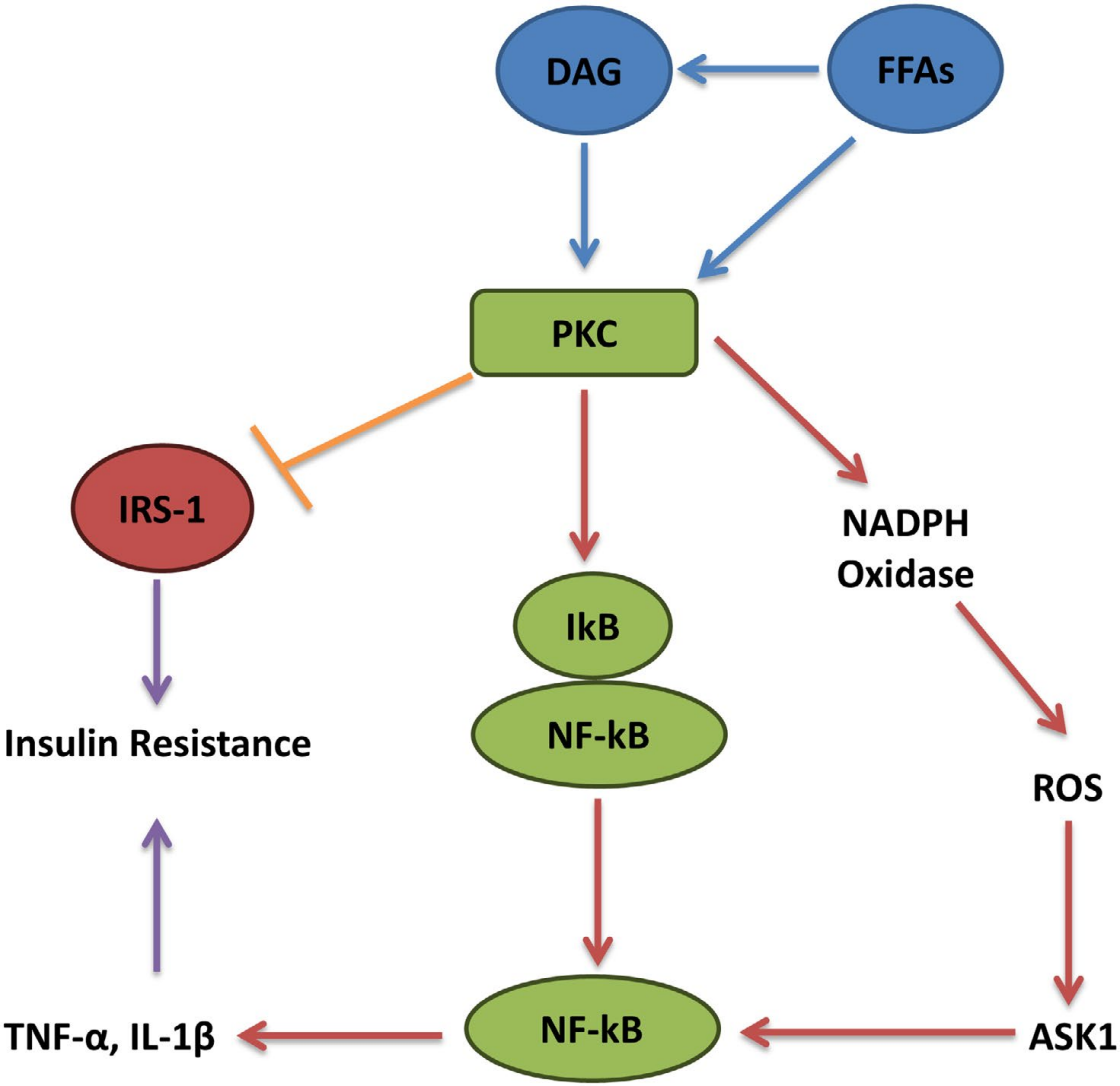
FFAs and DAG activate PKC, which in turn promotes insulin resistance via its effects on NF‐κB, NADPH oxidase and IRS‐1. ASK1, activation of apoptosis signal‐regulating kinase 1; DAG, diacylglycerol; FFAs, free fatty acids; IL‐1β, interleukin‐1 beta; IRS‐1, insulin receptor substrate‐1; NF‐κB, nuclear factor kappa‐light‐chain‐enhancer of activated B cells; PKC, protein kinase C; ROS, reactive oxygen species; TNF‐α, tumour necrosis factor‐alpha.

As discussed earlier, statins impair calcium channel function through the reduction of membrane cholesterol. Consequently, decreased membrane cholesterol disrupts the function of lipid domains within the plasma membrane, resulting in reduced localisation and impaired function of GLUT4 [8]. Thus, statins, particularly the lipophilic agents atorvastatin and simvastatin, have the potential to diminish insulin sensitivity via multiple mechanisms, including impairment of biogenesis and trafficking of GSV proteins, inhibition of insulin signalling, downregulation of key proteins such as GLUT4 and sortilin, and disruption of GLUT4 function. Within the cholesterol biosynthesis pathway, inhibition of HMG‐CoA reductase results in the accumulation of Acetyl‐CoA within cells. Because Acetyl‐CoA serves as a precursor for fatty acids, its accumulation promotes de novo fatty acid synthesis. Furthermore, statins activate the transcription factor sterol regulatory element‐binding protein 2 (SREBP‐2) through the reduction of intracellular cholesterol levels [67]. The accumulation of free fatty acids (FFAs) and metabolites of the fatty acid synthesis pathway, including diacylglycerol (DAG), results in the activation of serine–threonine kinases, such as protein kinase C (PKC) [18]. PKC modifies the structure of IRS‐1 through serine phosphorylation and prevents its tyrosine phosphorylation, thereby inhibiting insulin signalling [68]. Additionally, PKC activates NADPH oxidase, leading to increased production of superoxide anions [18]. It has been shown that reactive oxygen species (ROS) stimulate the activity of nuclear factor kappa‐light‐chain‐enhancer of activated B cells (NF‐κB) through activation of apoptosis signal‐regulating kinase 1 (ASK1) [69] (Figure 1). NF‐κB, as a transcription factor, upregulates a wide range of pro‐inflammatory factors such as cytokines [70]. Furthermore, PKC phosphorylates IκB, causing it to separate from NF‐κB and activating NF‐κB [71]. Tumour necrosis factor‐alpha (TNF‐α) and interleukin‐1 beta (IL‐1β) are pro‐inflammatory cytokines that are upregulated by NF‐κB [70]. These cytokines may act via paracrine, autocrine and endocrine mechanisms and, upon binding to their cell surface receptors, induce activation of IκB kinase (IKK). IKK then phosphorylates IκB, promoting its dissociation from NF‐κB, which results in NF‐κB activation and the progression of inflammation [72, 73]. IKK phosphorylates IRS‐1 at serine residues, which subsequently blocks its tyrosine phosphorylation and results in inhibition of the insulin signalling pathway. TNF‐α and IL‐1β not only activate IKK but also activate mitogen‐activated protein kinases (MAPKs) such as c‐Jun N‐terminal kinase (JNK), extracellular signal‐regulated kinase (ERK) and p38. These MAPKs, like IKK, inhibit insulin signalling by phosphorylating IRS‐1 on serine residues [74, 75, 76]. In a study reported by Kain et al., on skeletal muscle cells, it was proposed that simvastatin promotes insulin resistance via mechanisms dependent on FFAs. The study demonstrated that simvastatin upregulated fatty acid synthetase (FAS) and acetyl CoA carboxylase‐1 (ACC1) expression through activation of SREBP‐2. Furthermore, simvastatin elevated FFA levels by 80%, and this increase in FFAs induced by simvastatin led to impaired insulin‐stimulated glucose uptake [67]. Henriksbo et al. demonstrated that atorvastatin treatment of mouse adipose tissue attenuated AKT phosphorylation within the insulin signalling cascade in a manner dependent on IL‐1β. Moreover, atorvastatin activated p38 in adipose tissue stimulated with lipopolysaccharide [77].

Considering that studies have demonstrated that statins not only mitigate cholesterol but also lower triglyceride levels, it is highly probable that statins decrease metabolites involved in triglyceride synthesis, including FFAs and DAG. Moreover, statins have been demonstrated to diminish NF‐κB activity via inhibition of isoprenoid synthesis, resulting in decreased production of inflammatory mediators. Numerous studies have demonstrated the downregulation of inflammatory factors by statins, especially atorvastatin, simvastatin and rosuvastatin, in both in vitro and in vivo. Moreover, the antioxidant properties of statins have also been characterised [34, 78]. Thus, it is possible that activation of the FFA‐ and DAG‐dependent pathway by statins occurs with lesser intensity and only under certain conditions. Admittedly, only a limited number of preclinical studies challenge the anti‐inflammatory effects of statins in the process of increasing insulin sensitivity, which will be discussed later.

**FIGURE 2:**
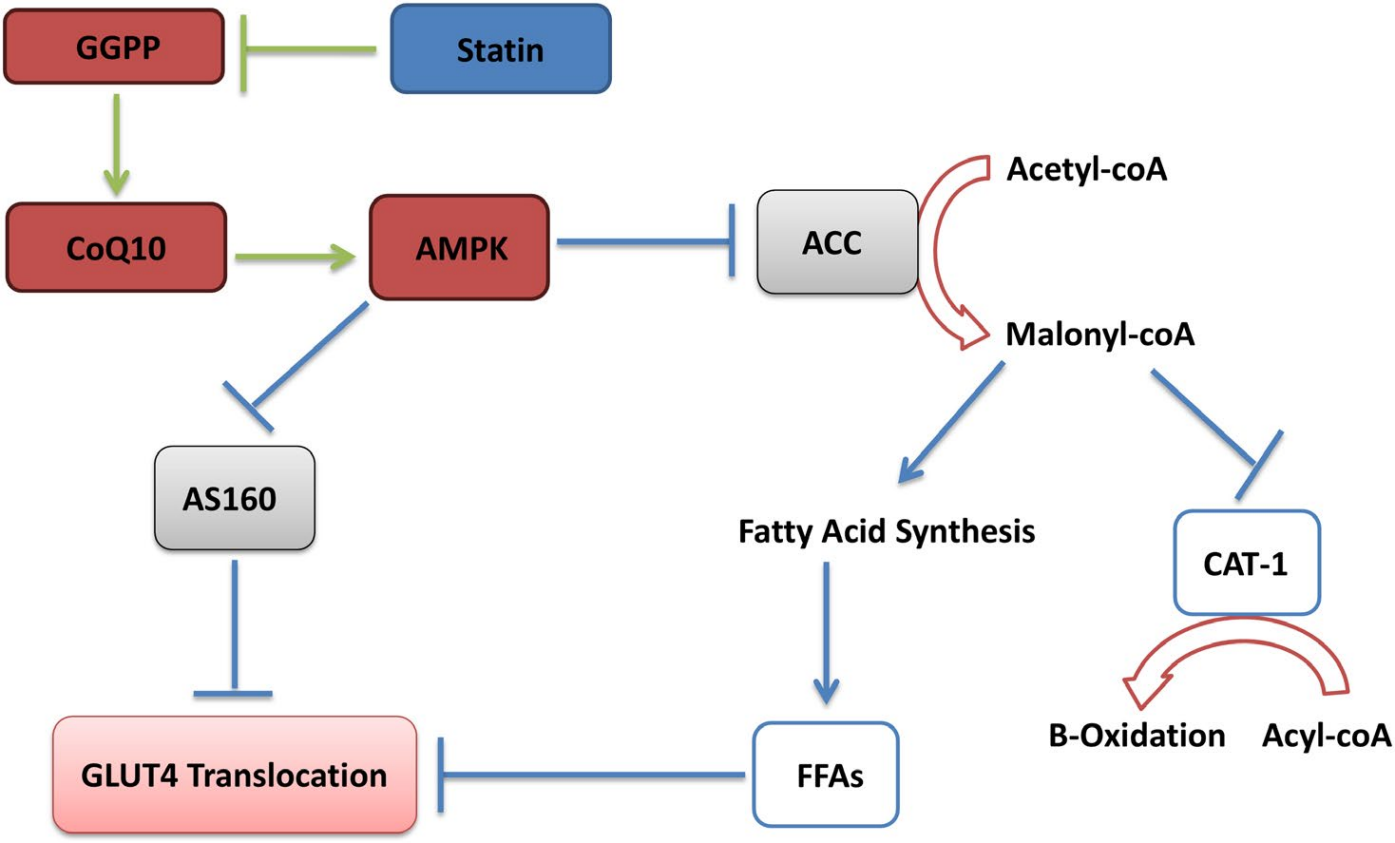
Statins inhibit GGPP synthesis, leading to decreased production or activity of coenzyme Q10 and AMPK. Through its effects on AS160 and on fatty acid synthesis and oxidation, AMPK mitigates cellular glucose uptake. ACC, acetyl CoA carboxylase; AMPK, 5′‐adenosine monophosphate–activated protein kinase; CAT‐1, carnitine acyltransferase‐1; GGPP, geranylgeranyl pyrophosphate.

As discussed earlier, statins reduce CoQ10 production by inhibiting GGPP formation, which leads to impaired insulin secretion. Lower CoQ10 levels not only disrupt insulin secretion but also cause insulin resistance. That is, reduced CoQ10 impairs fatty acid oxidation, causing lipids to build up inside cells and also mitigates the transport of GLUT4 vesicles to the cell membrane [79, 80]. CoQ10 induces the phosphorylation and subsequent activation of AMPK [81]. AMPK functions as an inhibitor of ACC within the fatty acid synthesis pathway. When active, ACC catalyses the conversion of Acetyl‐CoA to malonyl‐CoA. Malonyl‐CoA, in turn, acts as an allosteric inhibitor of carnitine acyltransferase‐1 (CAT‐1). CAT‐1 is involved in beta‐oxidation. It transfers Acyl‐CoA to carnitine at the outer mitochondrial membrane, forming acylcarnitine. Thus, AMPK promotes fatty acid oxidation by blocking malonyl‐CoA production [82]. AMPK can inactivate AS160 (a Rab GTPase‐activating protein) via phosphorylation, which maintains Rab proteins in their GTP‐bound conformation and promotes the transport of GSVs to the plasma membrane [83]. Thus, decreased levels and activity of CoQ10 may induce insulin resistance through reduction of AMPK activity (Figure 2).

In a study by Strey et al. treatment with atorvastatin (40 mg/day) for 6 weeks in patients with heart failure diminished CoQ10 levels [84]. Furthermore, Liu et al. demonstrated that treatment with atorvastatin (10 mg/day) for 5 months in hypercholesterolemic patients attenuated circulating CoQ10 levels [85]. Consistent with the previous study, Mabuchi et al. found the same result after 2 months of treatment [86]. Dohlmann et al. showed that administration of simvastatin (40 mg/day) in 64 patients, regardless of whether they experienced myalgia symptoms, did not produce a significant alteration in intramuscular CoQ10 levels [87]. Moreover, in a study by Shin et al., CoQ10 levels were not substantially altered following 2 months of simvastatin therapy (20–40 mg/day) in 76 hypercholesterolemic patients [88]. The lack of a significant effect on CoQ10 levels with simvastatin treatment may be attributable to its relatively weaker inhibitory activity on the cholesterol and isoprenoid synthesis pathway, in addition to its shorter half‐life relative to another lipophilic statin, atorvastatin. However, simvastatin remains a lipophilic statin which, owing to its hydrophobic characteristics and moderate half‐life, achieves higher concentrations within muscle cells. Therefore, depending on changes in experimental conditions, the effect on CoQ10 levels may vary. Accordingly, in a study by Trial et al., treatment with simvastatin (40 mg/day) for 2 weeks in 72 patients resulted in a significant decrease in plasma CoQ10 levels [89]. Scheffer et al. reported that administration of atorvastatin (10 mg/day) and simvastatin (40 mg/day) for 3 months, in patients with diabetes, significantly diminished circulating CoQ10 levels [90]. In a study by Larsen et al., simvastatin (10–40 mg/day) intake for a minimum of 12 months in hypercholesterolemic patients impaired glucose tolerance, mitigated insulin sensitivity index, reduced muscle CoQ10 content and diminished oxidative phosphorylation capacity in skeletal muscle biopsy specimens [79]. These results can be explained by simvastatin's lipophilic properties and the prolonged treatment period.

Moreover, in a study by Lamperti et al., the relationship between statin‐induced myopathy and muscular CoQ10 levels was examined. Patients were treated with simvastatin (*n* = 6), cerivastatin (*n* = 5), atorvastatin (*n* = 3) and rosuvastatin (*n* = 1). The findings demonstrated that statin treatment resulted in a mild, non‐significant decrease in muscle CoQ10 concentration [91]. The non‐significant changes in muscle CoQ10 levels were probably attributable to the small sample size, particularly the limited number of patients receiving statins that more effectively inhibit isoprenoid synthesis and possess longer half‐lives (atorvastatin and rosuvastatin). Notably, atorvastatin and simvastatin, due to their lipophilic properties, penetrate muscle cells more readily than rosuvastatin. Consequently, atorvastatin and simvastatin can achieve higher concentrations and exert greater effects on muscle CoQ10 levels compared to rosuvastatin. Nevertheless, as previously noted, rosuvastatin has a more potent inhibitory effect on the mevalonate pathway compared to atorvastatin. Because of its muscular and systemic toxicity, cerivastatin is no longer available for clinical use [20]. In a study by Chitose et al., treatment with atorvastatin (10 mg/day) and rosuvastatin (5 mg/day) for 6 months in patients with ST‐elevation myocardial infarction (STEMI) significantly attenuated serum CoQ10 levels [92]. Nevertheless, in a study by Toyama et al., treatment with atorvastatin (10–40 mg/day), in contrast to rosuvastatin (2.5–20 mg/day), substantially attenuated serum CoQ10 levels in patients with coronary artery disease [93]. Pitavastatin, despite its hydrophobic properties and cholesterol‐lowering effects, is less commonly used for the treatment of hypercholesterolemic patients due to its weaker cholesterol‐lowering effect compared to atorvastatin, as well as its shorter half‐life. In a study conducted by Kawashiri and colleagues, plasma CoQ10 levels in patients receiving low‐dose atorvastatin (20 mg/day) were significantly mitigated, whereas high‐dose pitavastatin (4 mg/day) had no significant effect on CoQ10 levels [94]. Furthermore, in a study reported by Keith and colleagues, patients with left ventricular dysfunction received lovastatin, pravastatin, atorvastatin and simvastatin at doses of 10–40 mg/day. The findings indicated that statin therapy did not cause a significant change in plasma, cardiac, or chest wall CoQ10 concentrations [95]. The explanation for this finding is that, although atorvastatin and simvastatin were used in this study, as previously noted, lovastatin and pravastatin exert weaker inhibitory effects on the mevalonate pathway and cholesterol synthesis relative to other statins. Furthermore, pravastatin, due to its hydrophilic properties, penetrates non‐hepatic cells to a lesser extent than other statins in this study. In addition, both lovastatin and pravastatin possess shorter half‐lives. In a study by Asping et al., treatment with simvastatin (80 mg/day) or pravastatin (40 mg/day) in healthy middle‐aged subjects for 2 weeks did not significantly change muscle CoQ10 levels [96]. Additionally, among the factors discussed, the short duration of patient follow‐up contributed to the absence of a significant change in CoQ10 levels. In a study by Bleske et al. involving 12 healthy volunteers, participants received atorvastatin (10 mg/day) and pravastatin (20 mg/day) for 1 month. The results showed no significant change in CoQ10 levels [97]. The absence of a substantial effect on CoQ10 levels with pravastatin has been addressed previously. Regarding atorvastatin, however, the lack of significant change in CoQ10 levels in this study can be attributed to the small sample size, the low dose of atorvastatin employed, and the short follow‐up period.

Phosphatase and tensin homologue (PTEN) is a negative regulator of insulin signalling, acting through inhibition of the phosphoinositide 3‐kinase (PI3K)/Akt pathway [98]. Statins have been demonstrated to increase both the expression and activity of PTEN. Teresi et al. showed that lovastatin induced upregulation of PTEN via peroxisome proliferator‐activated receptor‐gamma (PPAR‐γ) in the MCF‐7 breast cancer cell line [99]. Nevertheless, this cell line is not relevant to insulin sensitivity. Moreover, PPAR‐γ enhances insulin sensitivity through its antioxidant and anti‐inflammatory properties, as well as by stimulating GLUT4 expression and promoting the translocation of GSVs to the plasma membrane [100, 101, 102]. Thus, this finding raises contradictions with respect to the effect of lovastatin on reducing insulin sensitivity. In another study conducted on MCF‐7 cells, Teresi and colleagues showed that simvastatin, pravastatin, and fluvastatin led to dose‐dependent upregulation of PTEN [103]. Similar to other statins, in a study by Wu et al. on cardiac myxoma cells, atorvastatin mediated its therapeutic effects via enhancement of PTEN activity [104]. In a study published by Elbaset et al., pitavastatin attenuated renal injury in rats through enhancement of PTEN activity [105]. Furthermore, in a study by Birnbaum et al., treatment with rosuvastatin (10 mg/kg/day) for 30 days in mice promoted the development of diabetes through PTEN upregulation. Notably, partial knockdown of PTEN led to an improvement in rosuvastatin‐induced insulin resistance [106]. Nevertheless, additional studies are required to elucidate the relationship between statins and PTEN in insulin‐sensitive cells or in animal models.

**FIGURE 3:**
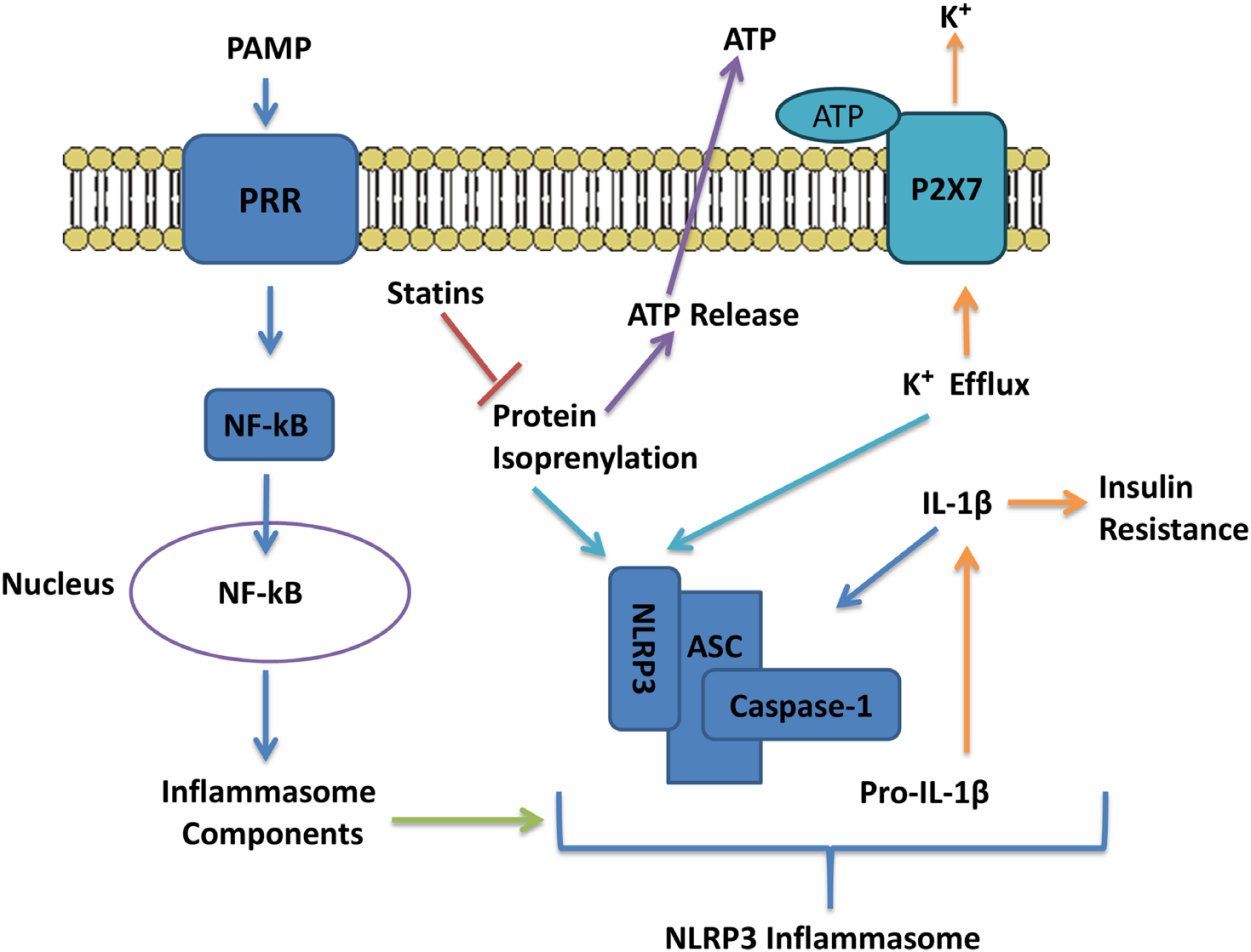
Effects of statins on insulin resistance via stimulation of the NLRP3 inflammasome. Statins inhibit protein isoprenylation, thereby promoting NLRP3 inflammasome activity. Additionally, inhibition of isoprenylation stimulates ATP release, which induces potassium efflux and subsequently activates the NLRP3 inflammasome. The activated NLRP3 inflammasome generates the mature, secreted form of IL‐1β. The activation of IL‐1β‐mediated signalling pathways subsequently promotes insulin resistance. Signalling pathways triggered by PAMPs and IL‐1β result in NF‐κB activation and upregulation of NLRP3 inflammasome components. ASC, apoptosis‐associated speck‐like protein containing a caspase recruitment domain; K ^+^, potassium; NLRP3, NACHT; LRR; and pyrin domain–containing protein 3; PAMP, pathogen‐associated molecular pattern; PRR, pattern recognition receptor.

An additional mechanism contributing to statin‐induced insulin resistance involves activation of the Nod‐like receptor family, pyrin domain containing 3 (NLRP3) inflammasome. Pathogen‐associated molecular patterns (PAMPs) trigger activation of specific receptors known as pattern recognition receptors (PRRs). Following stimulation of these receptors, NF‐κB induces transcription and increases the levels of NLRP3 inflammasome components and the non‐secretory form of IL‐1β (pro‐IL‐1β) [107, 108]. Nod‐like receptors (NLRs) are a class of PRRs. NLRP3, the most well‐characterised inflammasome, belongs to the NLR family and functions as a cytosolic sensor that responds to non‐microbial damage‐associated molecular patterns (DAMPs), including extracellular ATP [109, 110]. The NLRP3 inflammasome is composed of NLRP3, the adaptor protein ASC (apoptosis‐associated speck‐like protein containing a caspase recruitment domain), and caspase‐1. In response to various stimuli, the NLRP3 inflammasome becomes activated, leading to production of the mature, secreted IL‐1β [111] (Figure 3). As previously noted, IL‐1β contributes to the pathogenesis of inflammation and insulin resistance. Studies conducted on macrophages, cell lines and adipose tissue have demonstrated that statins activate the NLRP3 inflammasome, leading to enhanced IL‐1β secretion. Notably, fluvastatin has been shown to disrupt insulin signalling in adipocytes through increased caspase‐1 activity. Furthermore, simvastatin treatment in macrophages has been found to promote the processing of pro‐IL‐1β into its mature IL‐1β form [112, 113]. In a study reported by Henriksbo et al., fluvastatin promoted insulin resistance in murine adipocytes via activation of the NLRP3 inflammasome [114]. In a study conducted by Montero and colleagues on human peripheral blood mononuclear cells (PBMCs), fluvastatin‐induced activation of caspase‐1 was partially related to inhibition of protein prenylation. This activated caspase‐1 promoted the processing and secretion of bioactive IL‐1β. Moreover, in the presence of bacteria, fluvastatin synergistically and significantly increased IL‐1β release [115]. Furthermore, Montero‐Vega et al. reported that in fluvastatin‐treated PBMCs in the absence of immune challenge, there was significant production of NLRP3 inflammasome components. Consequently, the majority of monocytes/macrophages transformed into foam cells. Additionally, in bacterial‐exposed cultures treated with fluvastatin, ASC was released from foam cell nuclei, and co‐localisation of ASC with NLRP3 was increased. The combination of bacteria and fluvastatin markedly enhanced caspase‐1 activity. Based on these findings, fluvastatin‐mediated reduction of GGPP upregulated NLRP3 expression in foam cells. Therefore, it may be inferred that GGPP is involved in the downregulation of NLRP3 [116]. In a study conducted by Cui and colleagues on human THP‐1 cells, pitavastatin inhibited caspase‐1 activity, thereby suppressing mature IL‐1β release upon stimulation with pathogenic crystals, while having no effect on the expression levels of pro‐IL‐1β or pro‐caspase‐1. However, pitavastatin had no effect on NLRP3 expression. Exposure of cells to GGPP and FPP blocked the suppression of IL‐1β release caused by pitavastatin. Moreover, atorvastatin, fluvastatin and lovastatin did not significantly mitigate IL‐1β secretion under inflammatory conditions [117]. Saleh et al. demonstrated that treatment with rosuvastatin (10 mg/kg) and pitavastatin (0.8 mg/kg) for 1 month in rats with diabetic cardiomyopathy significantly diminished cardiac NLRP3 and IL‐1β levels [118]. Moreover, in a study by Wang et al. in rats, simvastatin ameliorated lung injury in hyperoxia‐induced bronchopulmonary dysplasia through decreased pulmonary expression of NLRP3, pro‐caspase‐1, cleaved caspase‐1, mature IL‐1β and ASC [119]. Lv and colleagues showed that in rat aortic endothelial cells, simvastatin improved hyperglycemia‐induced endothelial dysfunction by blocking the NLRP3 inflammasome [120]. Chen et al. demonstrated that atorvastatin treatment (10, 20, 40 mg/kg) in mice with intracerebral haemorrhage (ICH) attenuated neurological deficits via downregulation of NLRP3, cleaved caspase‐1 and IL‐1β [121]. In contrast, a study by Henriksbo et al. demonstrated that in mouse adipose tissue treated with atorvastatin and fluvastatin under lipopolysaccharide‐induced inflammatory conditions, NLRP3 inflammasome expression and IL‐1β secretion were increased. Moreover, atorvastatin and fluvastatin activated the NLRP3 inflammasome via p38 and impaired insulin signalling [77].

The studies mentioned showed contradictory effects regarding the association of statins with the NLRP3 inflammasome in various animal and cell models. However, overall, studies have shown that statins possess anti‐inflammatory properties that are linked to inhibition of GGPP and FPP synthesis [34]. However, according to the studies conducted, the effects of statins on the NLRP3 inflammasome in adipose tissue cells and macrophages may differ from those in other cells or tissues. Based on the published studies, several hypotheses exist regarding the mechanisms by which statins activate the NLRP3 inflammasome in adipose tissue. One possibility is that statins alone do not exert a significant effect on NLRP3 inflammasome activity; rather, they act synergistically with pathogens to enhance it. Specifically, PAMPs first bind to their receptors, activating NF‐κB and upregulating NLRP3 inflammasome components and pro‐IL‐1β. Subsequently, statins activate the NLRP3 inflammasome, resulting in increased IL‐1β secretion [113, 122]. Nevertheless, based on the aforementioned studies, statins also contribute to increased NLRP3 expression under inflammatory conditions [77, 116]. Another possibility is that activation of the NLRP3 inflammasome results from the inhibition of protein isoprenylation by statins [115, 116]. Statins enhance the release of intracellular ATP, a process that is probably mediated through inhibition of protein isoprenylation [123]. Extracellular ATP binds to a pattern recognition receptor (PRR) called P2X7 (purinergic receptor 7), leading to its activation. In adipose tissue macrophages, the P2X7 receptor functions as a ligand‐gated ion channel, inducing potassium efflux from the intracellular compartment. Decreased cellular potassium levels can induce activation of the NLRP3 inflammasome [113, 123]. In a study by Liao et al. using human THP‐1 cells, fluvastatin and lovastatin increased active caspase‐1 levels and IL‐1β secretion in LPS‐stimulated cells. In contrast, these statins had no effect on IL‐1β secretion in the absence of LPS. GGPP reversed statin‐induced caspase‐1 activation and IL‐1β secretion in LPS‐stimulated cells. Furthermore, statins induced caspase‐1 activation via stimulation of ATP release and P2X7 activation. Statins also weakly increased ROS levels, which were associated with increased caspase‐1 activity and IL‐1β secretion [123]. As previously noted, statins may impair mitochondrial function through decreasing CoQ10 levels. This mitochondrial dysfunction could result in a slight increase in ROS. Nevertheless, because statins possess considerable antioxidant properties, their net effect on ROS levels is minimal.

Contrary to the aforementioned studies, evidence has been shown that activation of the G‐proteins Rac1 and Cdc42 results in caspase‐1 activation. These G‐proteins require GGPP for their activation [123]. Thus, statins may suppress the NLRP3 inflammasome through reduction of G‐protein activity. This supports the earlier point that statins have anti‐inflammatory effects through GGPP inhibition [34]. Ying and colleagues reported that Rac1 activated the NLRP3 inflammasome via direct binding to NLRP3 and consequent exacerbation of diabetic nephropathy in mice [124].

There are several mechanisms that have been less investigated regarding their association with insulin resistance through statins. Statins can activate the pregnane X receptor (PXR) in hepatocytes. Activated PXR binds to phosphorylated serum/glucocorticoid‐regulated kinase 2 (SGK2) and facilitates its dephosphorylation by protein phosphatase 2C (PP2C). The nuclear complex formed by PXR and dephosphorylated SGK2 then upregulates the expression of phosphoenolpyruvate carboxykinase 1 (PEPCK1) and glucose‐6‐phosphatase (G6Pase) genes, resulting in increased hepatic glucose production [125]. Furthermore, statins increase gluconeogenesis in liver cells through stimulation of autophagy. In a study conducted by Wang and colleagues on mice and the HepG2 liver cell line, rosuvastatin, fluvastatin, pravastatin and atorvastatin led to increased mRNA and protein expression of PEPCK1 and G6Pase in HepG2 cells. Notably, autophagy inhibition attenuated statin‐induced PEPCK1 and G6Pase expression as well as gluconeogenesis. Statins promoted insulin resistance in high‐fat diet‐fed mice through stimulation of hepatic gluconeogenesis [126]. Studies have demonstrated that branched‐chain amino acids and lysine are associated with reduced insulin sensitivity. It was reported that simvastatin elevated the levels of acylcarnitines derived from valine, leucine, isoleucine and lysine, and this elevation was linked to the development of insulin resistance [127].

Disruption of preadipocyte differentiation into adipocytes, accompanied by impaired insulin signalling and decreased secretion of insulin‐sensitising hormones, results in the promotion of insulin resistance. For instance, mature adipocytes secrete adiponectin, which enhances insulin sensitivity. Additionally, adiponectin acts on pancreatic beta cells to inhibit their apoptosis. Statins can inhibit preadipocyte differentiation [128]. In a study by Khamis et al., exposure of human preadipocyte cell lines to lovastatin and atorvastatin resulted in impaired preadipocyte differentiation and diminished insulin signalling. Notably, statins attenuated the ability of insulin to activate AKT [129]. Moreover, Nakata et al. demonstrated that atorvastatin impaired the maturation of 3 T3‐L1 adipocytes, insulin action and GLUT4 expression, with these effects being reversed by mevalonate and GGPP supplementation. These findings highlight the importance of adipocyte maturation in enhancing insulin sensitivity [130]. In a study by Jakab et al., combined treatment with simvastatin and metformin suppressed lipid accumulation and differentiation of 3 T3‐L1 adipocytes. This observation presents a paradox, since, based on earlier discussions, inhibition of adipocyte differentiation mitigates adipocyte number, resulting in adipocyte hypertrophy and the promotion of inflammatory states and insulin resistance. Conversely, decreased lipid accumulation suppresses inflammation and insulin resistance, thereby mitigating the adverse effects of simvastatin and metformin. Admittedly, given the anti‐inflammatory effects of these two drugs, the detrimental consequences of reduced cell differentiation are minimal [131]. The mechanisms related to statin‐induced insulin resistance are shown in Table 2.

**TABLE 2:** Mechanisms responsible for decreased insulin sensitivity caused by statin therapy.

Suppression of cholesterol and isoprenoid production	Other roles
Impaired dolichol production	Activation of PXR activity and autophagy
Decreased activity of Rab‐4 and Rheb	Upregulation of the PEPCK1 and G6Pase genes
Disrupted function and localisation of insulin receptors and GLUT4	Increased expression and activity of PTEN
Impaired function of IRS‐1, AKT and GSK‐3β	Elevated levels of branched‐chain amino acids and lysine
Downregulation of the insulin receptor, IRS‐1, GLUT4, GSK‐3β, mTOR and sortilin	Decreased differentiation of preadipocytes
Disrupted translocation of GLUT4 to the plasma membrane	
Elevated levels of free fatty acids and their metabolites	
Decreased coenzyme Q10 levels	
Activation and increased expression of the NLRP3 inflammasome	
Enhanced IL‐1β secretion	
Slight elevation of ROS levels	

Abbreviations: AKT, protein kinase B; G6Pase, glucose‐6‐phosphatase; GSK‐3, glycogen synthase kinase 3; IL‐1β, interleukin‐1 beta; IRS‐1, insulin receptor substrate‐1; mTOR, mammalian target of rapamycin; NLRP3, nod‐like receptor family, yrin domain containing 3; PEPCK1, phosphoenolpyruvate carboxykinase 1; PTEN, phosphatase and tensin homologue; PXR, pregnane X receptor; ROS, reactive oxygen species.

### 3.3 Other Clinical Studies on the Association Between Statins and the Development of T2DM in Humans

The mechanisms associated with statin‐induced T2DM were discussed above, mainly based on preclinical studies, with a smaller contribution from clinical investigations. In this section, clinical studies that directly demonstrated the association between statins and T2DM were reviewed. Overall, evidence has been shown that statin users possessing one or more major risk factors for T2DM are at greater risk of developing the disease compared to those without such factors. Nevertheless, other contributing factors also play a role, as discussed earlier or as will be discussed later. Despite this, the benefits of statin therapy exceed the risk of incident diabetes, even among individuals who are at high risk for diabetes [8]. The first study to reveal a link between statin therapy and incident diabetes was the West of Scotland Coronary Prevention Study, which demonstrated that treatment with pravastatin lowered the risk of diabetes by 30% [24]. Subsequently, additional clinical studies were performed examining the relationship between statin therapy and the risk of incident diabetes. In a study by Abbasi et al. involving adults without established coronary heart disease or T2DM, 62% of participants were statin‐naïve, whereas 38% had prior statin exposure. Administration of atorvastatin (40 mg/day) for 10 weeks increased insulin resistance. No significant difference was observed in the effect of atorvastatin on insulin resistance between statin‐naïve individuals and those with prior statin exposure [132]. Despite the short follow‐up period and the fact that most individuals had not taken statins previously, considering the moderate dose of atorvastatin used and the previously discussed characteristics of this statin, the study provides evidence for atorvastatin‐induced insulin resistance. In contrast, in a case–control study by Andevari et al., statin‐naïve patients with T2DM were treated with low‐dose atorvastatin (20 mg/day) for 3 months. Compared to T2DM patients not receiving atorvastatin, the treated group exhibited lower circulating levels of HbA1c and fasting blood glucose. However, insulin levels were elevated in the atorvastatin‐treated group [133]. Furthermore, in a randomised clinical trial (RCT), Andevari et al. demonstrated that treatment with moderate‐dose atorvastatin (40 mg per day) for 3 months in statin‐naïve patients with T2DM mitigated HbA1c, fasting blood glucose and HOMA‐IR (Homeostatic Model Assessment for Insulin Resistance) [48]. These two previous findings showed that, despite the lipophilic properties and high cholesterol‐lowering capacity of atorvastatin, several factors may explain why atorvastatin did not worsen the diabetic condition. These factors include the patients having T2DM and taking glucose‐lowering medications, the short follow‐up period, the absence of prior statin exposure, and the administration of low and moderate atorvastatin doses. In fact, atorvastatin not only had no negative effect but also improved glycemic profile, insulin secretion and insulin sensitivity. In a study by Koh et al., patients with T2DM and metabolic syndrome received atorvastatin at doses of 10, 20, 40 and 80 mg/day (18–20 patients per dose group) for a period of 2 months. Atorvastatin therapy was associated with a significant elevation in HbA1c levels relative to baseline. This increase was consistently observed across the 20–80 mg dose range and was more pronounced compared to the 10 mg dose. Furthermore, insulin sensitivity was significantly mitigated following atorvastatin treatment [134]. Despite the short duration of follow‐up, the limited sample size, and the fact that some patients already had T2DM before treatment, given the explanations provided regarding atorvastatin properties and the fact that some patients were at the diabetes threshold, the drug induced glucose intolerance. Additional studies have investigated the relationship between statins and T2DM in patients who already had the disease. In a meta‐analysis by Zhou et al., the majority of study groups received atorvastatin, some received simvastatin or cerivastatin, and a small number of patients were treated with lovastatin or rosuvastatin. Overall, statin therapy did not significantly affect fasting plasma glucose, HbA1c, fasting insulin and HOMA‐IR. However, subgroup analysis revealed that atorvastatin had a substantial deleterious effect on HbA1c, whereas simvastatin was associated with an improvement in HbA1c [135]. Consistent with the above findings, statins did not exert adverse effects on insulin secretion or sensitivity in patients who had T2DM and were taking glucose‐lowering medications. It is possible, however, that long‐term statin administration, particularly high‐dose atorvastatin, in the context of poorly controlled diabetes despite pharmacological intervention may exacerbate glycemic parameters.

In a study published by Daido and colleagues, Japanese patients with T2DM and hypercholesterolemia received pitavastatin (2 mg/day) for 1 year. FBS levels significantly decreased in patients with BMI ≥ 25 compared to those with BMI < 25. Additionally, HbA1c levels significantly increased in the pitavastatin group among patients who had previously taken other statins and among those with BMI < 25, but the differences between the three groups were not significant. Notably, insulin resistance did not worsen following pitavastatin therapy [136]. These findings demonstrated that despite the moderate dose of pitavastatin, the long duration of follow‐up, and prior statin exposure in a subset of patients, pitavastatin did not exacerbate insulin resistance. This can be attributed to the aforementioned properties of pitavastatin and the fact that patients were receiving glucose‐lowering medications for their diabetic condition. These findings are consistent with the review by Agarwala et al., which concluded that pitavastatin has more neutral effects on incident diabetes compared to other statins [137]. Furthermore, Moutzouri et al. demonstrated that treatment with simvastatin (40 mg) or rosuvastatin (10 mg) for 3 months did not result in significant alterations in fasting plasma glucose, HbA1c, or the homeostasis model assessment of β‐cell function (HOMA‐β). In contrast, HOMA‐IR increased significantly [138]. These findings demonstrated that the use of a moderate‐dose lipophilic statin (simvastatin) and a low‐dose hydrophilic statin with high cholesterol‐lowering intensity (rosuvastatin) for a short duration in non‐diabetic patients, while having no significant effect on glycemic parameters or beta‐cell function, resulted in increased insulin resistance. This suggests that prolonged use of these statins may adversely affect glycemic control or even HOMA‐β via the exacerbation of insulin resistance. Consistent with this hypothesis, the study by Climent et al. reported that approximately 2 years of rosuvastatin therapy was associated with a 30% risk of new‐onset diabetes in hypercholesterolemic patients [139].

As discussed earlier, because pravastatin is hydrophilic and has weaker effects on blocking cholesterol synthesis, it may exert minimal influence on the exacerbation or development of T2DM, and could even lower the risk of incident diabetes. Unless, based on their metabolic characteristics, the patient has disorders that predispose them to T2DM [11]. In a meta‐analysis reported by Baker et al. on non‐diabetic patients, pravastatin therapy significantly enhanced insulin sensitivity [140]. As previously noted, beyond the intrinsic pharmacological properties of statins, both the dose and duration of therapy influence the association between statin use and incident diabetes. In a study by Zaharan et al. patients who had recently initiated statin therapy along with antidiabetic medications based on their lipid profile were examined. Treatment with rosuvastatin, atorvastatin and simvastatin was associated with an elevated risk of new‐onset treated diabetes, and this risk was significantly associated with higher doses and longer treatment duration. In contrast, fluvastatin and pravastatin did not substantially affect diabetes risk [141]. In a cohort study by Mansi et al. aimed at investigating the relationship between statin therapy and the risk of developing diabetes and its associated complications in non‐diabetic patients, over 90% of participants were treated with simvastatin or atorvastatin, whereas fewer than 10% received rosuvastatin or pravastatin. Patients were prescribed low, moderate, or high doses of statins, and the minimum follow‐up period was 3 months. The findings indicated that statin use was associated with a potential increase in the incidence of diabetes and its complications [142]. Moreover, in a meta‐analysis by Preiss et al. that included five RCTs, non‐diabetic patients received statin therapy for more than 1 year. The findings demonstrated that the risk of new‐onset diabetes was greater with intensive‐dose statin treatment (atorvastatin 80 mg or simvastatin 80 mg) than with moderate‐dose treatment (pravastatin 40 mg or simvastatin 20 mg) [143]. Consistent with previous findings, Sattar et al. reported in a meta‐analysis that statin therapy was associated with a modestly increased risk of incident diabetes. More specifically, atorvastatin, simvastatin and rosuvastatin were associated with a higher risk of T2DM compared to pravastatin and lovastatin. In this study, the majority of patients received long‐term statin therapy, and the highest rate of diabetes incidence occurred in older individuals [144]. In addition, in a cohort study by Carter et al. involving non‐diabetic individuals, after 3 years of follow‐up, atorvastatin, rosuvastatin and simvastatin were found to be associated with an elevated risk of new‐onset diabetes compared to patients treated with pravastatin (the reference drug for analysis). Conversely, no significant risk was observed with fluvastatin or lovastatin. The risk associated with rosuvastatin appears to be dose‐ and duration‐dependent [145]. In another cohort study, Cederberg et al. reported that statin use in non‐diabetic patients was associated with a 46% increased risk of incident T2DM, which was mediated by a 12% reduction in insulin secretion and a 24% reduction in insulin sensitivity. The effects of atorvastatin and simvastatin on mitigating insulin sensitivity and secretion, as well as on diabetes risk, were dose‐dependent and more pronounced than those of other statins. Furthermore, statins significantly increased 2‐h plasma glucose (2hPG) levels during oral glucose tolerance testing (OGTT) [146]. A systematic review of 16 systematic reviews and meta‐analyses by Singh et al. demonstrated that statin therapy was associated with an increased risk of new‐onset diabetes. Rosuvastatin and atorvastatin were more prominently linked to T2DM across many systematic reviews or meta‐analyses, whereas pravastatin and pitavastatin were associated with lower or no risk. High statin doses and advanced age were identified as important risk factors for diabetes [147]. Beyond the factors discussed in the interpretation of studies linking statins to T2DM, the findings of Bredefeld et al. indicated that, in addition to high statin doses, obesity and glycemic parameters close to the diabetes threshold in individuals were the main risk factors for statin‐induced T2DM [19]. In a cohort study conducted by Lichtenstein et al. involving patients with human immunodeficiency virus (HIV), each year of statin therapy was associated with a 14% increase in the incidence rate of diabetes. Moreover, the incidence of diabetes was elevated in individuals of older age and with higher body mass index. Notably, according to the study model, treatment duration also significantly contributed to the increased incidence of diabetes among participants [148].

## 4 Conclusions

Statins have been the most effective agents for managing hyperlipidemia and for both primary and secondary prevention of CVDs. Beyond their cholesterol‐lowering effects, statins also attenuate the incidence of complications associated with T2DM, including CVDs, cerebrovascular diseases, nephropathy, retinopathy and neuropathy, through their pleiotropic actions. The anti‐inflammatory and antioxidant effects of statins are part of the pleiotropic effects of these drugs, contributing to the reduction of T2DM incidence and its related pathological conditions [10, 18, 34].

Nevertheless, studies have demonstrated that statin use is associated with an elevated risk of new‐onset T2DM. Based on the studies reviewed above, the diabetogenic or diabetes‐exacerbating effects of statins are largely attributable to the inhibition of mevalonate pathway metabolites, including cholesterol and, more specifically, isoprenoids such as FPP and GGPP. Based on mevalonate pathway‐dependent and ‐independent mechanisms, both preclinical and clinical studies have demonstrated that statins, by modulating the levels or activity of various factors and proteins, including ATP, potassium and calcium channels, GLUT2, long‐chain acyl‐CoA, insulin receptor, IRS‐1, AKT, GLUT4, GSK‐3β, SREBP2, FAS, ACC1, CoQ10, PTEN, PPAR‐γ, NLRP3, IL‐1β, caspase‐1, ROS, PXR, PEPCK1, G6Pase and acylcarnitines, can impair insulin secretion and insulin sensitivity, thereby worsening glycemic control and promoting the development of T2DM. Nevertheless, differences exist among statins. Multiple factors influence the diabetogenic characteristics of statins. One such factor is their potency in inhibiting the mevalonate pathway; another is the extent of their lipophilicity. Atorvastatin and rosuvastatin exhibit greater potency in inhibiting the mevalonate pathway compared to other members of the statin class. In contrast, pitavastatin and pravastatin are less effective in suppressing this pathway. Regarding lipophilicity, simvastatin, atorvastatin, lovastatin, fluvastatin and pitavastatin are classified as lipophilic statins, whereas rosuvastatin and pravastatin are hydrophilic, with pravastatin being more hydrophilic than rosuvastatin. A third relevant factor is statin bioavailability, which is higher for hydrophilic statins, pitavastatin and fluvastatin than for other statins. The fourth factor is half‐life: rosuvastatin and atorvastatin possess a long half‐life; simvastatin and pitavastatin have a moderate half‐life; and other statins have a shorter half‐life.

Beyond the statin‐specific properties discussed above, several additional factors significantly influence the development of statin‐induced new onset T2DM. These include prior history of statin use, duration of follow‐up, statin dose, inflammatory conditions, cell type (e.g., adipose tissue cells and macrophages), patient age, blood pressure, obesity, history of diabetes and glycemic and lipid parameters approaching the diagnostic threshold for diabetes. Indeed, it has been demonstrated that statin users possessing one or more major T2DM risk factors are at higher risk of developing diabetes compared to those without such risk factors. However, the concurrent use of glucose‐lowering medications in patients with established T2DM mitigates the diabetogenic effects of statins. Despite their antioxidant and anti‐inflammatory effects, statins may, under specific circumstances delineated earlier, diminish insulin sensitivity through minor pro‐inflammatory and pro‐oxidant effects.

Despite the diabetogenic effects of statins, their use should not be discouraged. as the risk of CVDs is directly linked to elevated LDL‐C levels. So, keeping cholesterol low for long periods is clinically needed and results in reduced cardiovascular mortality. Despite the availability of several new lipid‐lowering agents, statins remain the first‐line treatment for the prevention of CVDs. Patients should be reassured that the benefits of statin therapy in preventing cardiovascular events greatly exceed the potential risk of abrupt elevations in plasma glucose. Glucose monitoring should be encouraged alongside continued statin treatment. Naturally, individuals can mitigate their risk of developing T2DM through lifestyle modifications, including dietary improvements and increased physical activity. Despite periodic monitoring of blood glucose or HbA1c, statin therapy should not be discontinued even if diabetes develops. Instead, consideration should be given to switching to a more metabolically favourable statin, administering statins at intermittent doses, or reducing the dose. However, if dose reduction or alteration of statin administration results in loss of cholesterol control, the original statin dose should be resumed and antidiabetic therapy added to the treatment regimen.

Based on the points discussed above, rosuvastatin emerges as the most suitable statin for the prevention of both microvascular and macrovascular complications for several reasons. First, it inhibits HMG‐CoA reductase to a greater degree than other statins. Second, it possesses high bioavailability and a prolonged half‐life. Third, as a hydrophilic statin, it exhibits few drug interactions, thereby reducing the risk of adverse effects such as new‐onset T2DM.

Bempedoic acid and ezetimibe represent therapeutic options that may be utilised in combination with reduced‐dose statins for patients who cannot tolerate statin therapy or experience statin‐related adverse effects, including diminished insulin secretion and sensitivity [39, 149, 150, 151]. Additionally, future preclinical and clinical research should examine how these drugs affect diabetes.

## Author Contributions

**Ali Nosrati Andevari:** investigation, conceptualization, writing – original draft, project administration, writing – review and editing. **Mohsen Koolivand:** writing – review and editing, validation.

## Funding

The authors have nothing to report.

## Conflicts of Interest

The authors declare no conflicts of interest.

## Data Availability

The data that support the findings of this study are available on request from the corresponding author. The data are not publicly available due to privacy or ethical restrictions.
